# Is the Impostor Phenomenon expressed in language? An LIWC analysis of textual self-descriptions

**DOI:** 10.3389/fpsyg.2025.1628389

**Published:** 2025-09-04

**Authors:** Kay Brauer, René T. Proyer

**Affiliations:** Department of Psychology, Martin Luther University Halle-Wittenberg, Halle (Saale), Germany

**Keywords:** Impostor Phenomenon, LIWC, personality, language, Clance Impostor Phenomenon Scale

## Abstract

The Impostor Phenomenon (IP) describes individual differences in self-perceptions of intellectual fraudulence. Based on the notion that personality traits are reflected in individual differences in language use, the literature provided initial evidence that the IP relates to language use. While earlier research was limited to job application letters, we expanded the study of the interconnectedness between the IP and language use by analyzing open self-descriptions (length limited to up to five sentences). We analyzed short textual self-descriptions by 325 participants with Linguistic Inquiry and Word Count (LIWC) software and examined their associations with self-reports of the IP. Contrary to earlier research, we found that the IP is unrelated to language use according to quantitative text analysis with the LIWC, except for using more words expressing anxiety (*r* = 0.22). Thus, our findings show that the IP is not robustly connected to language use in the domain of broad textual self-descriptions. We discuss implications for the interpersonal perception of the IP and discuss future directions to extend this line of research.

## 1 Introduction

The Impostor Phenomenon (IP; Clance, 1985) is a personality trait, with those showing high expressions (“Impostors”) being characterized by frequently doubting their intellectual abilities and feeling like a fraud although objective indicators of success such as grades, performance evaluations, and objective feedback are available (e.g., Brauer and Proyer, 2022). Impostors attribute their performance to external factors such as luck and chance and discount their abilities (Brauer and Proyer, 2022, 2025b). Accordingly, those high in IP fear being “exposed” as intellectual fraudsters and experience reduced positive psychological functioning and inclinations to depressiveness, anxiety, and fear of negative evaluation (e.g., Brauer and Wolf, 2016; Chrisman et al., 1995; Vergauwe et al., 2015). While there is increasing interest in research on the IP (Stone-Sabali et al., 2023), most studies have focused on the study of data derived from self-report questionnaires and, to our knowledge, only two studies had investigated the IP using other data sources: Ibrahim et al. (2023) examined the interpersonal perception of the IP using other ratings and Brandt et al. (2024) studied how the IP relates to language use in job application materials. We expanded Brandt and colleagues’ line of research on the interconnectedness between the IP and language by testing how the IP relates to word use in short textual self-descriptions.

The notion that individual differences in personality are connected to how people express and describe their emotions, thoughts, and behaviors has been one of the foundations of taxonomically oriented trait psychology (e.g., Allport and Odbert, 1936; Baumgarten, 1933). One important channel for expressing stable patterns of personality traits is language. For example, the sedimentation hypothesis assumes that descriptors of relevant personality traits are manifested and communicated through language (Allport and Odbert, 1936). Since the introduction of automated quantitative text analysis, which allows one to analyze individual differences in language use (Pennebaker and King, 1999), there is increasing interest and knowledge about the role of language use in psychology (Jackson et al., 2022). The interconnectedness of language and personality traits has received major interest (e.g., Čolović et al., 2023). This approach allows one to understand individual differences from a perspective beyond standard self-report questionnaires, showing how traits are expressed and perceived through language (e.g., Fast and Funder, 2008). Understanding connections between personality traits and language often extends beyond the individual and contributes to understanding how personality plays a role in social relationships.

Pennebaker and King (1999) introduced a software called *Linguistic Inquiry and Word Count* (LIWC) which analyzes text-based language (e.g., transcripts of interviews or self-descriptions) and matches the words with about 80 pre-defined categories describing formal (e.g., word count), grammatical (e.g., use of pronouns), and psychological (e.g., affect) features. Since the introduction of LIWC, there have been numerous studies that examined how personality traits are expressed in language (see Chung and Pennebaker, 2018, and Tausczik and Pennebaker, 2010, for overviews). Typical effect sizes between LIWC-derived variables and personality traits are of medium size—on average 0.23 (Hirsh and Peterson, 2009).

The focus on language use has been extended from analyzing associations with traits to examining whether others utilize information from language use (so-called linguistic cues) to infer expressions of traits. For example, Brauer and Proyer (2020) conducted two studies in which they analyzed the linguistic correlates of three narrower traits, namely, dispositions toward ridicule and being laughed at (i.e., fear of being laughed at, joy in being laughed at, and joy in laughing at others; Ruch and Proyer, 2009). They asked participants to provide short self-descriptions about themselves and then tested how accurately unacquainted judges inferred the expressions in the three dispositions. They found that language use played a role, as it contained valid information about the dispositions and helped explain the agreement between self- and others’ reports of personality traits. For example, their meta-analysis of findings across studies showed that while fear of being laughed at related to using less words describing laughter, those who enjoyed being laughed at more often used the words “laugh” and “laughter” when describing themselves. Moreover, judgments of the dispositions accurately correlated with such linguistic cues, suggesting that others utilize language use as indicators of personality expressions. While the latter example contained a correct utilization of linguistic cues by judges, findings have also expanded knowledge on which linguistic cues are *inaccurately* used by judges. For example, judges perceived others as high in fear of being laughed at when they utilized words describing general worries although the cue was invalid, as language use of worry-related words is independent from self-reports of fear of being laughed at. The analysis of language use as indicators of personality traits has not been limited to learning more about those who produce linguistic information, but it has also considered how others perceive their personality traits on the basis of linguistic cues. Hence, language use also relates to how people perceive others and accordingly translates to consequences such as relationship formation (e.g., Ireland et al., 2011). Overall, the study of textual information has contributed to our understanding of how trait-relevant information is expressed through language and taken up by others for their inferences on traits such as the broad Big Five traits, depressiveness, sense of power, and narrower traits like cheerfulness playfulness, and dispositions toward ridicule and being laughed at (Borkenau et al., 2016; Brauer and Proyer, 2020, 2025a; Körner et al., 2024; Lau et al., 2021; Rodriguez et al., 2010).

The IP is characterized by feelings of intellectual fraudulence and has consequences such as low self-esteem, inclinations to anxiousness of being exposed as an impostor, and barriers to career development, as well as concerns about self-presentation in real-life and online contexts (e.g., Chrisman et al., 1995; Ibrahim et al., 2024; Neureiter and Traut-Mattausch, 2016, 2017). One might argue that the IP might go along with individual differences in language use that might reflect these insecurities, but there is hitherto only limited knowledge about the IP and how it is expressed in language. To our knowledge, only Brandt et al. (2024) addressed the question of whether IP expressions relate to language use. The authors analyzed self-reports of IP assessed with the Impostor-Profile (IPP30; Ibrahim et al., 2022) and job application materials (i.e., cover letter and curriculum vitae [CVs]) of 70 participants, using LIWC software. In short, they found that higher IP did correlate with writing longer sentences (i.e., words per sentence; *r* = 0.33) and the use of more causation words (*r* = 0.28) across the materials. Also, the IP did go along with using more words that expressed orientation to rewards (*r* = 0.32) and less work-related words (*r* = −0.29). These findings provided initial insights into how the IP can be expressed in language, but Brandt et al. discussed that these findings were hitherto limited to the field of work and text materials in the context of job applications and required extension to other life domains (e.g., some LIWC categories such as anxiety showed expectedly low base rates), as well as noting the comparatively small sample size that might affect the stability of the findings.

### 1.1 The present study

In this study we aimed to expand Brandt et al.’s (2024) line of research by investigating participants’ general textual self-descriptions as a basis for the analysis of language use in relation to the IP. The prior research had shown that textual self-descriptions are suited to learn more about the interconnectedness between personality and language use (e.g., Borkenau et al., 2016; Brauer and Proyer, 2025a; Körner et al., 2024; Lau et al., 2021; Proyer and Brauer, 2018; Rodriguez et al., 2010), and we followed this line of research. In accordance with Brandt et al., we used the LIWC approach for a systematic quantitative text analysis of the self-descriptions in relation to IP self-reports.

First, we examined whether findings regarding language use in job application materials would generalize to self-descriptions. Thus, we investigated whether the previously observed associations between the IP and the use of longer sentences, causation words, reward-related language, and work-related terms also persist when self-descriptions are analyzed. Second, the literature had shown that the IP is characterized by self-presentation strategies, and it could be argued that those high in IP tend to compare themselves with others; thus, we expected to find a positive relation between the IP and the use of more comparison words. Third, we expected to find more anxiety-related words in the self-descriptions of those with high IP expressions when considering that Impostors fear being exposed as an intellectual fraud, as they have internalized that their achievements and successes are based on luck and chance (Brauer and Proyer, 2022, 2025b). Finally, considering that Impostors report frequent negative affect and depressiveness (Brauer and Wolf, 2016; Chrisman et al., 1995; Vergauwe et al., 2015), we assumed that the IP would coincide with the use of words that express sadness. Since sadness and anxiety are subcategories of the higher-order category “negative emotion words,” we expected to find a positive association between the IP and the use of more negative emotion words.

## 2 Methods

### 2.1 Sample

We analyzed the data of 325 participants, of which 61.2% identified as women, 36.3% as men, 1.5% as non-binary/third gender, and three participants did not indicate their gender. Their mean age was 25.9 years (*SD* = 7.4). The majority were students (89.3%), 8.0% were working professionals, four participants were engaged in a voluntary social year, three were retired, and two were job-seeking. The educational status was high, according to participants’ highest earned degree since 67.4% held the high-school diploma qualifying one to attend university (“Abitur”), 23.7% held a university degree, 5.2% had completed vocational training, and the remainder held a regular high school diploma.

Power analyses in G*Power (Faul et al., 2009) showed that our sample allowed for the detection of the average correlation effect size for findings based on LIWC software in personality research (*ρ* = 0.23; Hirsh and Peterson, 2009), with 98.9% power when assuming a 5% type-I error rate and two-tailed tests of statistical significance. In addition, a test of sensitivity showed that the sample size was sufficient to detect correlations ≥ 0.15, with 80% power. Moreover, our sample size exceeded thresholds needed for estimating stable correlation estimates (Schönbrodt and Perugini, 2013).

### 2.2 Instruments

We assessed the Impostor Phenomenon (IP) with the *German-language Clance Impostor Phenomenon Scale* (GCIPS; Brauer and Wolf, 2016; English original by Clance, 1985). The GCIPS includes 20 items (e.g., “It’s hard for me to accept compliments or praise about my intelligence or accomplishments”) and participants provide their responses on a 5-point rating scale (1 = *never*; 5 = *always*). There is robust evidence for the reliability and validity of the original and German-language version of the measure (Brauer and Proyer, 2025b; Brauer and Wolf, 2016; see also Mak et al., 2019, for an overview regarding the reliability and validity). We computed the total score, yielding an internal consistency of 0.91 in the present study.

We analyzed the participants’ language use in their self-descriptions with the LIWC-22 software (Boyd et al., 2022), using the German-language dictionary (Meier et al., 2019). As suggested by Meier et al. (2019), we corrected the spelling in the text data to ensure that the LIWC software correctly matched the words from the self-descriptions with the internal dictionary. The German-language version of the LIWC has frequently been used to study language use (e.g., Brandt and Herzberg, 2020; Brauer et al., 2022; Hartnagel et al., 2025; Körner and Schütz, 2023), and there is robust evidence for the reliability and validity of the German LIWC dictionary (see Meier et al., 2019). In accordance with the literature which used the LIWC to analyze associations with personality traits, we corrected the correlations with the IP scores for the LIWC’s reliability (0.59; see Hirsh and Peterson, 2009).

### 2.3 Procedure

We advertised our study via social media and leaflets on-campus by providing a link to an online questionnaire (hosted by www.soscisurvey.de). We did not provide financial compensation for participation, but psychology students could earn course credit. When entering the online study, participants provided informed consent and were then asked to provide demographic information and then a self-description, using up to five sentences. There were no further instructions for this task, and it was noted that there were no guidelines or expectations for the self-descriptions. This procedure had been successfully used in prior research involving analyses of personality traits and quantitative language analysis (e.g., Brauer and Proyer, 2020, 2025a; Körner and Schütz, 2023; Lau et al., 2021). After completing the writing task, participants completed the GCIPS (Brauer and Wolf, 2016) and were debriefed. We conducted this research in line with the Declaration of Helsinki and the ethical guidelines of the German Psychological Association. This type of research in Germany is exempt from approval by an ethics committee.

## 3 Results

The descriptive statistics of the GCIPS (*M* = 59.62, *SD* = 13.84) showed slightly higher expressions (Cohen’s *d* ≤ 0.43) than previously reported for German-speaking samples (Brauer and Wolf, 2016). We observed no deviation from a normal distribution (skewness and kurtosis ≤ |0.35|). As in earlier research, the GCIPS scores did not relate to gender (*r* = 0.06, *p* = 0.298), but there was a trend for an association of younger age with higher expressions of the Impostor Phenomenon (IP; *r* = −0.19, *p* = 0.001).

The self-descriptions were comprised of between four and 150 words (*M* = 43.1, *SD* = 22.7, *Mdn* = 41), which is comparable to other studies that used this approach (e.g., Brauer and Proyer, 2025a; Körner et al., 2024). The recognition of the words by the LIWC dictionary was high, with *M* = 89.9% (*SD* = 13.8%, *Mdn* = 91.7%).

Our main analysis which examined the associations between the LIWC and GCIPS scores showed that the IP was widely independent from language use, as reflected in the frequency of word categories covered by the LIWC. All correlation effect sizes were ≤ 0.14 (see the Supplementary materials for all coefficients; 77 correlations [83.7%] < |0.10|), except for the use of more anxiety-related words (*r* = 0.22, 95% confidence interval [0.12, 0.33], *p* < 0.001). The latter met our expectations. Findings from the domain of job application letters (Brandt et al., 2024) did not generalize to self-descriptions—we did not find associations with causation (*r* = 0.05, [−0.06, 0.16]), words per sentence (*r* = 0.03, [−0.08, 0.14]), reward motivation (*r* = 0.00, [−0.11, 0.11]), or work-related words (*r* = −0.07, [−0.18, 0.04], *p*s ≥ 0.208). Furthermore, we found the expected positive associations with the use of negative emotion words and comparison words (*r*s = 0.12, [0.01, 0.23], *p*s ≤ 0.030), but effect sizes were small. A closer inspection of the negative emotion category showed that the subcategories of anger (*r* = 0.08, [−0.03, 0.19]) and sadness (*r* = −0.05 [−0.16, 0.06], *p*s ≥ 0.150) were not robustly related to the IP. Hence, it can be assumed that the association with negative emotion words was based on the anxiety subcategory and was, thus, negligible despite statistical significance.

## 4 Discussion

With this study we aimed at extending knowledge about individual differences in the Impostor Phenomenon (IP) by extending initial knowledge about its interconnectedness with language use. Building on Brandt et al. (2024), who addressed this question recently regarding the professional domain by investigating the language use in CVs and job application letters, we examined the generalizability of their findings to general textual self-descriptions. Using a paradigm frequently used to study associations between language use and personality traits (Brauer and Proyer, 2025a; Körner and Schütz, 2023; Lau et al., 2021; Proyer and Brauer, 2018) and a comparatively large sample, we analyzed textual self-descriptions with the LIWC approach (Boyd et al., 2022; Pennebaker and King, 1999).

Our findings showed that the IP is widely independent from language use by means of LIWC software (i.e., word counts in about 80 categories which cover grammatical and psychological characteristics). We found one robust correlation which showed that higher IP related to the use of more anxiety-related words (e.g., “worry,” “fear,” “afraid,” and “nervous”), with a medium effect size. This fits well with the theoretical conception of the IP (Clance, 1985; Ibrahim et al., 2022) and empirical findings (e.g., Brauer and Wolf, 2016; Chrisman et al., 1995; Vergauwe et al., 2015), showing that high IP expressions are characterized by inclinations to fear of negative evaluations, generalized anxiousness, and fear of being exposed as an intellectual fraud. While we found this association in our self-descriptions, there was no association in job application materials in Brandt et al. (2024). This would support Brandt et al.’s notion that expressing anxiety is context-dependent and is avoided in professional contexts like when preparing job application letters, although it is expressed in less formal settings, such as expressions in self-descriptions.

While our expectations were met concerning Impostors using more words describing social comparisons and negative emotions in self-descriptions, these findings must be interpreted cautiously. First, the effect sizes were small, and the practical implications are likely negligible. Second, the association with the higher-order category was driven by the finding on anxiety-related words because associations with the other subcategories of anger and sadness were negligible. Thus, the finding on negative emotion is likely based on the overlap with the anxiety subcategory and does not hold unique value. Third, while the category of comparison words is still available in the German-language dictionary (Meier et al., 2019), it has been excluded from the English-language dictionary since Boyd et al. (2022) highlighted that this category has been found to show low internal reliability.

Finally, Brandt et al.’s (2024) finding that Impostors’ produced more words per sentence, expressed reward motivation, and used work-related and causation words did not replicate in our study. Several reasons might account for this finding. Again, differences in settings might play a role. While job application letters are localized in professional domains and highly formalized, our approach allowed participants to freely express themselves. Also, job application letters are not anonymous, whereas our approach allowed the participants anonymity, which might correlate with levels of expression. Furthermore, perhaps a difference is also that we asked participants to limit their writing to a maximum of five sentences, which might have led to more succinct writing. While this would not reflect in words per sentence, it could also be noted that cover letters in job applications are also typically limited in length, thus, requiring a comparatively succinct writing style. Moreover, past studies differed regarding the assessment of the IP, with Brandt et al. using the IPP30 (Ibrahim et al., 2022), whereas the present study used the GCIPS (Brauer and Wolf, 2016; Clance, 1985). Although there is robust overlap between the measures, with *r* = 0.78 according to Ibrahim et al. (2021), it is possible that differences between measures play a role.

Another difference between Brandt et al.’s (2024) and our findings might be based on motivation: While the language materials analyzed by Brandt et al. were likely prepared with a certain level of motivation that relates to being selected for a job, the writing task in our study was not related to a high-stakes situation. Therefore, differences in participants’ motivation may have influenced the degree of specificity of the self-descriptions. Cross-replication of Brandt et al.’s and our findings is desirable to clarify the role of the assessment instrument for potential differences.

Taken together, our study showed that the IP is widely unrelated with language use in self-descriptions with regard to quantitative measures of word use. Our findings have several implications. One conclusion is that the findings on associations between the IP and language use from the domain of work (i.e., CVs and job application letters; Brandt et al., 2024) do not generalize to self-descriptions. This highlights the domain-dependent specificity of language use and might have implications for the interpersonal perceptions of the IP. For example, Borkenau et al. (2016) showed that the accuracy of judgments about Big Five traits can depend partly on the life domain people write about. They asked participants to write about five domains (i.e., hobbies, family, friends, academic studies, and future plans) and found that, for example, extraversion can be inferred more accurately when people write about friends in comparison to judgments per future plans.

One implication is that it might be good news that the IP is independent from language use in terms of word use as covered by LIWC software since this implies that IP tendencies are likely difficult to infer from text-based information. Accordingly, our findings might help to explain Impostors’ preferences for engaging in online communication and using social network sites (Ibrahim et al., 2024), when considering that the IP is mostly independent from language use in textual self-descriptions. Similarly, there is evidence that students are anxious to communicate in person, according to data collected in second-language courses (Brauer et al., 2023). If Impostors feel more comfortable when using online communication and self-presentation online, an additional implication is that this might help them find and foster relationships with friends, colleagues, or partners through online-based dating portals and social networks.

Another implication (and future direction) is that it would be interesting to learn more about whether people can infer expressions of the IP from self-descriptions or job application materials. As discussed, our findings would imply that the lack of expression in language use should go along with lower accuracy in judging the IP from text-based materials, as no linguistic cues are available apart from using anxiety-related words. The prior research had shown that the IP can be accurately perceived among knowledgeable others (Ibrahim et al., 2023), but it is still unclear which information people use when inferring expressions of the IP in others. Self-descriptions have been successfully used in the study of the interpersonal perception of narrow traits (e.g., playfulness, cheerfulness, and dispositions toward ridicule and being laughed at; Brauer and Proyer, 2020; Lau et al., 2021; Proyer and Brauer, 2018) and allow one to investigate which linguistic cues others use for their judgments. It would be interesting to examine whether judgments of the IP are also unrelated to LIWC categories or whether judges utilize linguistic cues erroneously. Extending the study of the interconnectedness between the IP and language to other perceptions would contribute new knowledge to the field.

### 4.1 Limitations and future directions

This study’s findings must be interpreted with its limitations in mind. First, our study was limited to German-speakers. While this allowed us to compare our findings with Brandt et al.’s (2024) with regard to the language but also by using the same LIWC dictionary (Meier et al., 2019), generalizability to other languages than German is needed to ensure that findings hold across languages. Meier et al. (2019) provided evidence for the high agreement between the English and German LIWC dictionaries but noted that differences in base rate are language-specific and, thus, empirical verification of cross-language invariance is desirable. Second, we utilized self-reports of the IP as our criterion. While this is the standard approach of the field, and considering evidence on the reliability and validity of the GCIPS (Brauer and Proyer, 2025b; Brauer and Wolf, 2016), one must assume that self-reports are confounded with subjective biases that might affect the estimation of correlations through method variance. Third, although our approach to using self-descriptions is a standard in the field, we note that asking participants to write no more than five sentences provides standardization and comparability of the texts on the one hand but might also set boundaries on the length of the essays on the other hand. Finally, the LIWC approach is a quantitative measure that provides frequency analyses of word use. While this approach is reliable and provides important insights into language use beyond formal and grammatical categories, it cannot yet provide a comprehensive and holistic analysis of meaning and is still limited in its ability to extract qualitative features of texts beyond word frequencies.

In conclusion, our findings contribute to the knowledge about the IP as an individual difference variable, as we built on the prior research (Brandt et al., 2024) regarding its interconnectedness with language. To our knowledge, this was one of the few studies that examined the IP by using an alternative source of information beyond self-reports, and we hope that our findings stimulate further research on the IP in relation to language use and its interpersonal perceptions.

## Data Availability

The datasets presented in this study can be found in online repositories. The names of the repository/repositories and accession number(s) can be found at: https://osf.io/4yqcs/.
