# Correction: Is the Impostor Phenomenon expressed in language? An LIWC analysis of textual self-descriptions

**DOI:** 10.3389/fpsyg.2025.1702036

**Published:** 2025-10-03

**Authors:** Kay Brauer, René T. Proyer

**Affiliations:** Department of Psychology, Martin Luther University Halle-Wittenberg, Halle (Saale), Germany

**Keywords:** Impostor Phenomenon, LIWC, personality, language, Clance Impostor Phenomenon Scale

In the published article, there was an error in the *p*-value regarding the finding on the association between IP scores and anxiety-related words that falsely indicated *p* = 0.001 instead of *p* < 0.001.

A correction has been made to section **3 Results**, *Paragraph 3*. The corrected paragraph is shown below.

“Our main analysis which examined the associations between the LIWC and GCIPS scores showed that the IP was widely independent from language use, as reflected in the frequency of word categories covered by the LIWC. All correlation effect sizes were ≤ 0.14 (see the Supplementary materials for all coefficients; 77 correlations [83.7%] < |0.10|), except for the use of more anxiety-related words (*r* = 0.22, 95% confidence interval [0.12, 0.33], *p* < 0.001). The latter met our expectations. Findings from the domain of job application letters (Brandt et al., 2024) did not generalize to self-descriptions—we did not find associations with causation (*r* = 0.05, [−0.06, 0.16]), words per sentence (*r* = 0.03, [−0.08, 0.14]), reward motivation (*r* = 0.00, [−0.11, 0.11]), or work-related words (*r* = −0.07, [−0.18, 0.04], *p*s ≥ 0.208). Furthermore, we found the expected positive associations with the use of negative emotion words and comparison words (*r*s = 0.12, [0.01, 0.23], *p*s ≤ 0.030), but effect sizes were small. A closer inspection of the negative emotion category showed that the subcategories of anger (*r* = 0.08, [−0.03, 0.19]) and sadness (*r* = −0.05 [−0.16, 0.06], *p*s ≥ 0.150) were not robustly related to the IP. Hence, it can be assumed that the association with negative emotion words was based on the anxiety subcategory and was, thus, negligible despite statistical significance.”

The original version of this article has been updated.

