# Supplementary material for: Is the Impostor Phenomenon expressed in language? An LIWC analysis of textual self-descriptions
Source: Front Psychol. 2025 Sep 4;16:1628389. doi: 10.3389/fpsyg.2025.1628389 (PMC12443818; doi:10.3389/fpsyg.2025.1628389)
Supplement: Supplementary file 1 [file Table_1.DOCX]

**Electronic Supplementary Materials**

**ESM A**

*Correlations Between the German Clance Impostor Phenomenon Scale Scores (IP) and Word Use Extracted with the Linguistic Inquiry and Word Count (LIWC)*

| LIWC Category | IP | 95% Confidence Interval |
| --- | --- | --- |
| Summary Variables |  |  |
| Word Count | -.04 | [-.15, .07] |
| Analytical thinking | .01 | [-.10, .12] |
| Clout | -.01 | [-.12, .10] |
| Authentic | -.01 | [-.12, .10] |
| Emotional Tone | .02 | [-.09, .13] |
| Words per Sentence | .03 | [-.08, .14] |
| Words > 6 Letters | .00 | [-.11, .11] |
| Linguistic Dimensions |  |  |
| Total function words | -.06 | [-.17, .05] |
| Pronouns (All) | -.04 | [-.15, .07] |
| Personal Pronouns (All) | -.03 | [-.14, .08] |
| 1st Person Singular | -.01 | [-.12, .10] |
| 1st Person Plural | -.08 | [-.19, .03] |
| 2nd Person | -.06 | [-.17, .05] |
| 2nd Person Plural | -.06 | [-.17, .05] |
| 3rd Person (All) | -.02 | [-.13, .09] |
| 3rd Person Singular | -.05 | [-.16, .06] |
| 3rd Person Plural | -.08 | [-.19, .03] |
| Impersonal pronouns | .00 | [-.11, .11] |
| Articles | -.13* | [-.24, -.02] |
| Prepsositions | .10 | [-.01, .21] |
| Auxiliary verbs | -.02 | [-.13, .09] |
| Adverbs | .05 | [-.06, .16] |
| Conjunctions | -.03 | [-.14, .08] |
| Negations | -.08 | [-.19, .03] |
| Common verbs | -.04 | [-.15, .07] |
| Common adjectives | .04 | [-.07, .15] |
| Comparison words | .12* | [.01, .23] |
| Interrogation | -.01 | [-.12, .10] |
| Numbers | .03 | [-.08, .14] |
| Quantities | .06 | [-.05, .17] |
| Psychological Processes |  |  |
| *Drives* | -.11* | [.00, .22] |
| Affiliation | -.07 | [-.18, .04] |
| Achievement | -.08 | [-.19, .03] |
| Power | .05 | [-.06, .16] |
| *Cognitive Processes* | .03 | [-.08, .14] |
| Insight | .00 | [-.11, .11] |
| Causation | .05 | [-.06, .16] |
| Discrepancy | .07 | [-.04, .18] |
| Tentative | .11* | [.00, .22] |
| Certainty | -.09 | [-.20, .02] |
| Differentiation | .05 | [-.06, .16] |
| *Affect* | .08 | [-.03, .19] |
| Positive Emotions | .02 | [-.09, .13] |
| Negative Emotions | .12* | [.01, .23] |
| Anxiety | .22*** | [.12, .33] |
| Anger | .08 | [-.03, .19] |
| Sadness | -.05 | [-.16, .06] |
| Swear words | .02 | [-.09, .13] |
| *Social* | .05 | [-.06, .16] |
| Family | -.04 | [-.15, .07] |
| Friends | -.09 | [-.20, .02] |
| Female references | .11* | [.00, .22] |
| Male references | -.02 | [-.13, .09] |
| Expanded Dictionary |  |  |
| *Lifestyle* |  |  |
| Work | -.07 | [-.20, .02] |
| Leisure | -.03 | [-.14, .08] |
| Home | -.16** | [-.27, -.05] |
| Money | -.08 | [-.19, .03] |
| Religion | -.03 | [-.14, .08] |
| *Biological Processes* | .08 | [-.03, .19] |
| Body | .03 | [-.08, .14] |
| Health | .11* | [.00, .22] |
| Sexuality | .01 | [-.10, .12] |
| Death | -.09 | [-.20, .02] |
| *Motives* |  |  |
| Reward | .00 | [-.11, .11] |
| Risk | -.01 | [-.12, .10] |
| *Perceptual Processes* | .13* | [.02, .24] |
| Seeing | .05 | [-.06, .16] |
| Hearing | .13* | [.02, .24] |
| Feeling | .01 | [-.10, .12] |
| Relativity  (sum of space, time, motion) | -.04 | [-.15, .07] |
| Motion | -.16** | [-.27, -.05] |
| Space | .00 | [-.11, .11] |
| Time | .02 | [-.09, .13] |
| Time_past | -.04 | [-.15, .07] |
| Time_present | -.02 | [-.13, .09] |
| Time_future | .12* | [.01, .23] |
| *Conversational* |  |  |
| Informal words | .01 | [-.10, .12] |
| Netspeak | .12* | [.01, .22] |
| Assent | .02 | [-.09, .13] |
| Fillers | -.04 | [-.15, .07] |
| Punctuation |  |  |
| Punctuation (All) | -.04 | [-.15, .07] |
| Period | .02 | [-.09, .13] |
| Comma | .04 | [-.07, .15] |
| Colon | -.06 | [-.17, .05] |
| Semicolon | .04 | [-.07, .15] |
| Exclamation | -.02 | [-.13, .09] |
| Questionmark | .03 | [-.08, .14] |
| Dash | .07 | [-.04, .18] |
| Quote | .02 | [-.09, .13] |
| Parentheses | .09 | [-.02, .20] |
| Other punctuation | .02 | [-.09, .13] |

*Note.* *N* = 325. **p* < .05. ** *p* < .01. *** *p* < .001. Two-tailed. In accordance with Hirsh and Peterson (2009), correlations were corrected for attenuation concerning the LIWC’s reliability of α = .59. Correlations controlled for age and gender.
